# Refining Shoulder Diagnostics: A Technical Note on Scapular Physical Examination

**DOI:** 10.1002/atn2.70203

**Published:** 2026-07-31

**Authors:** Farah Selman, Nicholas Peter James Perry, Diego J. Restrepo, David Coleman, Joaquin Sanchez‐Sotelo

**Affiliations:** ^1^ Department of Orthopedics Balgrist University Hospital Zurich Switzerland; ^2^ Department of Orthopedic Surgery Mayo Clinic Rochester Minnesota U.S.A.; ^3^ Department of Orthopedic Surgery Naval Medical Center San Diego San Diego California U.S.A.; ^4^ Media Support Services Mayo Clinic Center for Surgical Support Operations (MCSSO) Rochester Minnesota U.S.A.

## Abstract

Normal scapulothoracic function relies on a delicate balance among several periscapular muscles and is essential for shoulder motion. Disturbance in this balance can lead to abnormal motion, which can impair shoulder function, leading to pain and discomfort. A thorough scapular examination requires an accurate physical assessment and the ability to identify pathologies, such as muscle paralysis (e.g., trapezius or serratus anterior) and hyperactivity (e.g., pectoralis minor). Overall, a comprehensive approach to the physical examination is critical in accurately diagnosing and effectively treating shoulder and scapular pathologies. The purpose of this technical note is to provide a concise, standardized methodology for performing a comprehensive scapular examination, focusing on key clinical tests, anatomical landmarks, and motion patterns.

## Graphical abstract

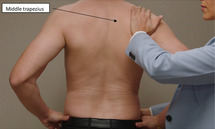

**VIDEO 1:** Demonstration of key components of the scapular physical examination described in this Technical Note. Video content can be viewed at https://doi.org/10.1002/atn2.70203.

The scapula plays an essential role in shoulder mechanics, providing a stable base for glenohumeral movement while facilitating force transmission across the shoulder complex. Aberrations in scapular motion, often termed “scapular dyskinesis”^1^ and “scapulothoracic abnormal motion,” can be associated with various common shoulder pathologies, including rotator cuff tears,^2^ impingement,^2^, ^3^, ^4^, ^5^ and instability,^2^, ^3^ leading to shoulder pain.^6^ Since the evaluation and treatment of scapular pathologies are still developing, many surgeons have not had the opportunity to develop an in‐depth understanding of the scapular physical examination.

This limited focus on the scapula in standard shoulder examinations often overlooks subtle but clinically significant alterations in scapular movement and muscular function, which can be crucial indicators of underlying shoulder pathologies. Therefore, a detailed and systematic approach to scapular examination is essential in diagnosing, treating, and monitoring scapulothoracic abnormal motion and associated pathologies.

The purpose of this technical note is to provide a concise, standardized methodology for performing a comprehensive scapular examination, focusing on key clinical tests, anatomical landmarks, and motion patterns. Emphasis is placed on reliable techniques that are both practical in the clinical setting and supported by evidence for diagnostic accuracy. By establishing a clear framework for scapular assessment, this technical note seeks to enhance diagnostic precision and facilitate a more targeted approach to therapeutic intervention in patients presenting with shoulder and periscapular complaints.

## PHYSICAL EXAMINATION MANEUVERS

The scapula should be examined with an uncovered torso, from several angles and most importantly from posteriorly. Before specific scapular examination, the surgeon should conduct a standard shoulder examination to better understand global shoulder function and identify common shoulder conditions. Additionally, the cervical spine should be assessed to rule out potential cervical pathologies affecting shoulder function.

### Clavicle, Acromioclavicular, and Sternoclavicular Joints

The scapula glides over the chest wall and builds with the acromioclavicular joint,^7^ the sternoclavicular joint, and various periscapular muscles of the shoulder girdle.^8^ Instability of the acromioclavicular or sternoclavicular joints can affect scapular function and should therefore be examined through palpation and corresponding stress tests like Cross‐Body,^9^ O’Brien,^9^ and arm traction test.^10^

Clavicular shortening, assessed by side‐to‐side comparison, can lead to scapular internal rotation and anterior tilt, presenting as excessive protraction at rest.^11^

### Muscle Control and Motion

Table 1 summarizes the muscle groups involved in scapular motion, depending on the direction of movement.

**TABLE 1:** Primary Muscles Involved in Scapular Motion

**Movement**	**Primary Muscles Involved**
Scapular retraction, proximation to the midline	Middle trapezius, rhomboids
Scapular protraction	Serratus anterior, pectorals minor
Scapular elevation	Levator scapulae, rhomboid minor and major, upper trapezius
Scapular depression	Lower trapezius, pectoralis minor, latissimus dorsi
Arm elevation	Serratus anterior, upper and lower trapezius
Arm depression	Rhomboids, levator scapulae, pectoralis minor

*Note*: This table summarizes the key muscular contributors to various scapular movements, including retraction, protraction, elevation, depression, and arm motion.

### Pectoralis Minor

Borstad et al.^12^ showed that a shortened pectoralis minor can mimic scapular motion seen in shoulder impingement, suggesting it may contribute to subacromial pathology. Shortening or contracture of the pectoralis minor can be assessed by measuring the distance between the tip of the coracoid at the location of the joint between the 4^th^ rib and the sternum and comparing it with the opposite side.

### Resting Position of the Scapula and Scapulothoracic Rhythm

When viewing the patient from the posterior, the resting position of the scapula can be observed, and scapular rhythm can be assessed with repeated active flexion and abduction. Prominence of the inferior pole or the medial edge of the scapula or frank scapulothoracic abnormal motion can be appreciated. Measuring the distance between the spine midline and the medial border of the scapula can be helpful, especially in patients with paralysis of the trapezius, where the affected side will be further away from the midline than the normal side.

### Trapezius Strength

**FIGURE 1:**
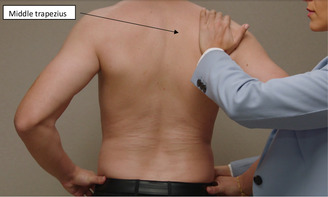
Examination of middle trapezius strength. In order to examine the right shoulder middle trapezius strength, the patient is viewed from the posterior while resting both hands resting on the hip. In this position, scapular retraction is tested against manual resistance. This position helps isolate the middle trapezius from the rhomboids. The arrow shows the region of the middle trapezius.

Upper trapezius strength can be tested by asking the patient to shrug the shoulders against resistance. The strength of the middle trapezius and rhomboids can be assessed by resisted scapular retraction. The trapezius can be isolated from the rhomboids by positioning the arm in slight abduction, the hands resting on the hip, and repeated assessment of scapular retraction against resistance (Figure 1, Video 1).

Middle trapezius strength can also be assessed with scapular retraction against resistance with the patient prone in approximately 90° of flexion.

Similarly, the strength of the lower trapezius can be assessed with resisted retraction with the arm in approximately 120° of flexion.

### Scapular “Flip”

Patients with paralysis of the trapezius will have exaggerated prominence of the medial border of the scapula during resisted external rotation, which is considered a positive scapular flip test.

### “Boxer Punch”

The bower punch test helps to evaluate the strength of the serratus anterior muscle. With the arm at 90° of forward flexion and the elbow almost fully extended, the patient is asked to push against resistance. In addition to weakness, the medial border of the scapula may also lift off the chest wall in the resisted punching position.

### Scapular Assistance Test

**FIGURE 2:**
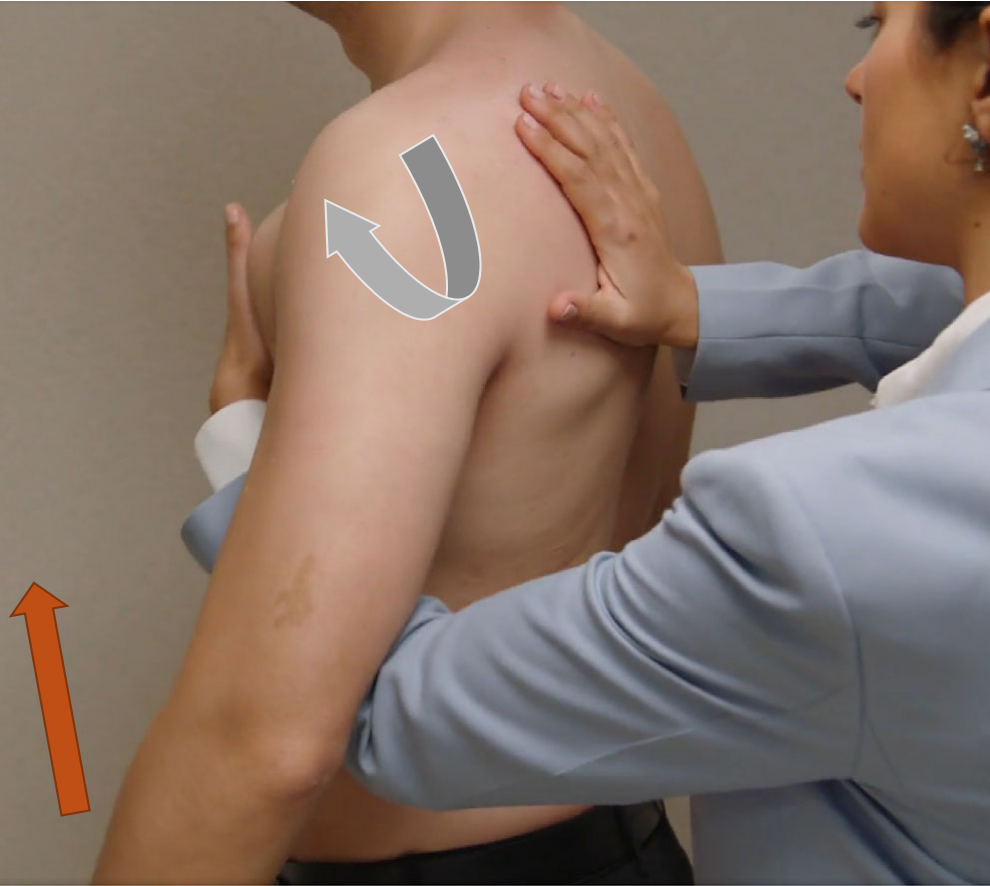
Scapular assistance test. In order to examine the left shoulder, the examiner stands behind the patient and applies manual upward rotation and posterior tilt to the left scapula (right hand, gray arrow) during active arm elevation by the patient (left arm, orange arrow). This maneuver aims to simulate normal scapular motion and assess whether assisted scapular movement reduces pain or improves range of motion, which would indicate dysfunctional scapular mechanics such as serratus anterior hypoactivity or early facioscapulohumeral dystrophy.

The scapular assistance test aims to determine whether supporting the scapula on the chest would lead to improved function. It is particularly useful for the evaluation of serratus palsy, serratus hypoactivity, and the early stages of facioscapulohumeral dystrophy. The examiner manually applies an upward rotation and a posterior tilt force to the scapula during arm elevation (Figure 2). If scapular assistance causes a reduction in pain or improved range of motion, this test confirms that poor scapular kinetics are contributing to shoulder dysfunction.

### Wall Push‐Up

Serratus anterior fatigue can be assessed by asking the patient to perform repetitive, slow push‐ups against a wall. If the medial border of the scapula lifts off, this is suggestive of serratus anterior muscle weakness.

### Shoulder Flexion Resistance Test

**FIGURE 3:**
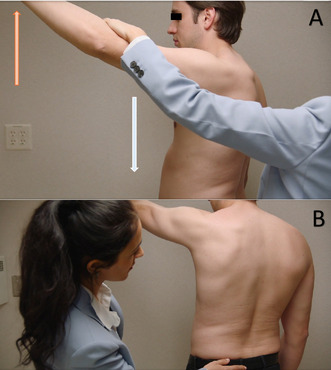
SFRT. (A) While examining the left shoulder, the examiner views from the posterior on the affected side. The patient performs resisted shoulder flexion at approximately 100°. (B) View from posterior. Scapula observation. The test is conducted in multiple angles (30°, 60°, and 100°) to assess serratus anterior function and differentiate between hypoactivity and paralysis. Prominence of the inferior scapular border at lower angles that resolves by 100° suggests serratus anterior hypoactivity, whereas persistent scapular prominence at all angles indicates serratus anterior paralysis. (SFRT, shoulder flexion resistance test.)

The shoulder flexion resistance test^13^ is designed to assess the function of the serratus anterior and pectoralis minor muscles. It can also help distinguish between serratus anterior hypoactivity versus paralysis. Increased prominence of the inferior scapular boarder during resisted flexion testing at 30° and 60° which resolves at 100° of flexion is suggestive of serratus anterior hypoactivity with possible associated pectoralis minor hyperactivity or contracture (Figure 3). On the contrary, in patients with serratus anterior palsy, the prominence of the inferior scapular border is present at all angles of forward flexion.

Since the pectoralis minor and the upper parts of the serratus anterior share similar origins, both contribute to scapular movement at lower degrees of flexion. Theoretically, at 100° of flexion, the influence of the pectoralis minor is minimized, allowing for a more isolated assessment of serratus anterior function.

### Scapular Snapping

With the examiner's hand over the affected area, the patient is asked to repeat the arm motion that causes the scapular snapping symptoms. The examiner attempts to feel or hear the snapping phenomena reported by the patient.

## DISCUSSION

This technical note and its accompanying video present a practical and standardized framework to conduct a comprehensive clinical evaluation of the scapula by assessing position, muscle function, and dynamic motion. A systematic evaluation of scapular motion is essential for the accurate diagnosis and management of shoulder dysfunction and pain.^6^ Despite its biomechanical significance,^2^, ^4^, ^5^, ^14^ examination of the scapula is often minimized or neglected during routine shoulder assessments. Therefore, this technical note has attempted to address a critical gap in musculoskeletal examination routines.

Scapulothoracic abnormal motion is not a diagnosis in itself, but rather a manifestation of underlying pathological and often neuromuscular impairments. Anatomically, the scapula functions as a dynamic base for upper limb motion. Disruptions in the normal scapulothoracic rhythm, due to clavicular shortening,^11^ pectoralis minor contracture,^12^ or neuromuscular insufficiency, can compromise glenohumeral function and lead to secondary pathologies such as subacromial impingement or instability.^15^, ^16^ As Sciascia and Kibler^15^ emphasize, scapular dyskinesis should be approached as a physical impairment within a broader clinical context, rather than as an isolated diagnosis.

The examination techniques presented, such as the scapular assistance test, flip sign, and shoulder flexion resistance test, enable clinicians to link altered scapular motion to patient symptoms and identify potential underlying causes. The findings from Borstad et al.^12^ and Lohre et al.^13^ underscore the relevance of distinguishing between muscular imbalance and paralysis. Specifically, the shoulder flexion resistance test has been shown to provide excellent diagnostic accuracy in identifying serratus anterior dysfunction, outperforming the traditional wall push‐up test.^13^

Unfortunately, the integration of 3‐dimensional scapular motion in clinical models remains challenging due to the scapula's deep anatomical position. Although advanced motion capture or biomechanical modeling (e.g., bone‐pin or ellipsoid‐based systems) provide research‐level insights, their practical implementation remains limited.^17^ Thus, a thorough physical examination remains the most feasible tool in routine practice.

It is important to remember that scapular abnormalities must be interpreted in the context of the broader kinetic chain. The scapula plays a pivotal role in energy transfer from the trunk and lower extremity to the arm. Therefore, dysfunction may not only be localized but also influenced by distant impairments along the chain. Targeted rehabilitation should focus on restoring neuromuscular control, not just isolated strength, especially in cases of functional dyskinesis.^15^

In summary, the scapular examination techniques outlined in this technical note offer a reliable and efficient method to assess key elements of scapular function. When incorporated into standard shoulder assessments, they have the potential to significantly enhance diagnostic accuracy and guide appropriate treatment strategies for a range of shoulder pathologies.

## DISCLOSURES

The authors (F.S., N.P.J.P., D.J.R., D.C., J.S‐S.) declare the following financial interests/personal relationships which may be considered as potential competing interests: F.S. reports a relationship with Mayo Clinic in Rochester. N.P.J.P. reports a relationship with Arthroscopy: The Journal of Arthroscopic and Related Surgery that includes: board membership. D.J.R. reports a relationship with Mayo Clinic in Rochester. D.C. reports a relationship with Mayo Clinic in Rochester. J.S‐S. reports a relationship with Stryker Orthopaedics, Exatech, and Acumed LLC that includes: consulting or advisory; reports a relationship with Oxford University Press; reports a relationship with American Shoulder and Elbow Surgeons that includes: board membership; reports a relationship with PrecisionOS Technology that includes: equity or stocks; reports a relationship with Orthobullets that includes: equity or stocks; reports a relationship with PSI that includes: equity or stocks; reports a relationship with Elsevier.

## FUNDING

Open access publishing facilitated by Universitat Zurich, as part of the Wiley ‐ Universitat Zurich agreement via the Consortium Of Swiss Academic Libraries.
